# The Identification of Novel Mutations in ATP-Dependent Protease ClpC1 Assists in the Molecular Diagnosis of Obscured Pyrazinamide-Resistant Tuberculosis Clinical Isolates

**DOI:** 10.3390/microorganisms13061401

**Published:** 2025-06-16

**Authors:** H. M. Adnan Hameed, Cuiting Fang, Zhiyong Liu, Yamin Gao, Shuai Wang, Xinwen Chen, Nanshan Zhong, Htin Lin Aung, Jinxing Hu, Tianyu Zhang

**Affiliations:** 1 State Key Laboratory of Respiratory Disease, Joint School of Life Sciences, Guangzhou Institutes of Biomedicine and Health, Chinese Academy of Sciences, Guangzhou Medical University, Guangzhou Chest Hospital, Guangzhou 510530, China; adnan@gibh.ac.cn (H.M.A.H.); wshuai1@163.com (S.W.); 2 China-New Zealand Joint Laboratory of Biomedicine and Health, Guangzhou Institutes of Biomedicine and Health, Chinese Academy of Sciences, Guangzhou 510530, China; htin.aung@otago.ac.nz; 3 Guangdong-Hong Kong-Macau Joint Laboratory of Respiratory Infectious Diseases, Guangzhou 510530, China; 4 University of Chinese Academy of Sciences, Beijing 100049, China; 5 Guangzhou National Laboratory, Guangzhou 510005, Chinananshan@vip.163.com (N.Z.); 6 State Key Laboratory of Respiratory Disease, National Clinical Research Centre for Respiratory Disease, Guangzhou Institute of Respiratory Health, The First Affiliated Hospital of Guangzhou Medical University, Guangzhou 510120, China; 7 Department of Microbiology and Immunology, School of Biomedical Sciences, University of Otago, Dunedin 9016, New Zealand

**Keywords:** *Mycobacterium tuberculosis*, pyrazinamide, drug resistance, *clpC1*, clinical isolates, molecular diagnosis

## Abstract

Pyrazinamide (PZA) is a key component of tuberculosis treatment, with drug resistance (PZA^R^) primarily related to *pncA* mutations. However, discordance between phenotypic resistance and conventional *pncA*-based molecular diagnostics challenges diagnostic accuracy. This study investigates discrepancies between phenotypic and genotypic resistance profiles among *Mycobacterium tuberculosis* (Mtb) clinical isolates. Fifty-three Mtb isolates from Guangzhou Chest Hospital were tested for PZA resistance using the BACTEC MGIT 960 system and PZase activity assay. Thirty-one phenotypically PZA^R^ strains were genetically assessed by Sanger sequencing of PZA^R^-associated customary genes. Five *pncA*-wild-type PZA^R^ strains were investigated through whole-genome sequencing. ClpC1P1P2 activity was evaluated by proteolytic degradation assay. Notably, 26/31 of the PZA^R^ strains harbored mutations in *pncA* and/or its upstream region, aligning PZase activity and phenotypic profiles. However, five PZA^R^ strains lacked *pncA* mutations. The WGS of five discordant strains revealed four novel mutations (Gly58Ser, Val63Ala, Ala567Val, and Pro796Leu) across ClpC1 domains. Incorporating *clpC1* mutations improved molecular diagnostic sensitivity and accuracy from 48.3% and 69.8% (*pncA* alone) to 100%. This is the first report from southern China that identifies novel *clpC1* mutations in wild-type *pncA* PZA^R^ Mtb isolates. Our findings underscore the limitations of *pncA*-targeted diagnostics and support the integration of WGS and *clpC1* analysis in molecular diagnostics to prevent false-negative diagnoses and improve clinical outcomes.

## 1. Introduction

Tuberculosis (TB), caused by *Mycobacterium tuberculosis* (Mtb), remains one of the leading infectious causes of death worldwide and a major public health concern. Each year, about 10 million new TB cases and ~1.5 million deaths due to TB are reported around the globe [1]. The increasing prevalence of multidrug-resistant (MDR) and extensively drug-resistant (XDR) TB presents a serious threat to global TB control efforts. These resistant forms are associated with prolonged treatment, poorer outcomes, and limited therapeutic options.

Pyrazinamide (PZA), an important first-line anti-TB drug, is a key component of treatment regimens for both drug-susceptible and MDR TB. Its unique sterilizing activity against non-replicating, persistent Mtb significantly shortens treatment duration and reduces relapse rates [2]. Recent studies, including our evaluation of the sudapyridine + clofazimine + TB47 (SCT) regimen combined with PZA (SCTZ) in a well-established murine TB model, demonstrated superior efficacy compared to the standard BPaL regimen. Notably, SCTZ achieved complete sterilization within two months without relapse [3]. Despite its efficacy, the global rise in PZA resistance is concerning, with over 60% of MDR-TB cases exhibiting concurrent resistance to PZA [4].

The emergence of drug resistance in Mtb is mainly caused by a combination of factors [5,6,7,8,9,10]. These include genetic mutations within the bacteria, which can alter drug targets and render the antibiotics ineffective [10]. Diagnostic inaccuracies such as false-negative results, delayed detection of resistance, or misclassification of susceptibility can lead to inappropriate or ineffective therapy. This exerts selective pressure on Mtb populations, fostering the survival and amplification of drug-resistant strains, which potentially increases the risk of MDR and XDR TB [1,8,11]. In addition, undiagnosed or misdiagnosed cases can facilitate the transmission of resistant strains within communities, worsening the public health burden [6]. Accurate and timely diagnostics, including molecular assays such as whole-genome sequencing (WGS), are essential for detecting resistance-associated mutations and guiding effective treatment regimens [7,8,9]. Moreover, the use of inadequate therapeutic regimens, through incorrect combinations, dosages, or durations, further contributes to the development of resistance [5]. Finally, patient non-adherence to prescribed treatments enables the persistence and evolution of resistant strains [7,8,9]. Addressing these factors is crucial for effective TB control.

Although PZA’s mechanism of action is complex and not fully elucidated, it is known to target multiple pathways essential for the survival of persistent Mtb. PZA is a prodrug that needs to be converted to its active form, pyrazinoic acid (POA), by pyrazinamidase (PZase), encoded by the *pncA* gene. Mutations in *pncA* or its regulatory region, leading to PZase inactivation or reduced expression, are the most common mechanism of PZA resistance [12,13]. However, other genes have also been implicated in PZA resistance. These include *rpsA*, encoding ribosomal protein S1, an essential component of the trans-translation system that binds POA and is critical under stress conditions [14]; *panD*, encoding aspartate decarboxylase involved in CoA biosynthesis [15]; and *Rv2783c*, a bifunctional enzyme implicated in (p)ppGpp metabolism and nucleic acid hydrolysis [16].

Moreover, some earlier studies have highlighted the role of ClpC1, an ATP-dependent protease, in PZA resistance [17]. However, data on ClpC1 and its association with PZA resistance in clinical isolates of Mtb remain limited, underscoring the need for updated investigations to refine potential resistance markers for molecular diagnosis. Notably, most molecular diagnostic methods for detecting PZA resistance focus solely on mutations in *pncA*. However, PZA^R^ Mtb strains lacking mutations in customary genes such as *pncA*, *rpsA*, *panD*, and *Rv2783c* have been reported in clinical studies [12], suggesting the presence of obscured resistance mechanisms that compromise the accuracy of molecular diagnostic tools.

Moreover, the genotyping of Mtb clinical isolates provides valuable epidemiological insights into drug-resistant TB. The Beijing genotype (Lineage 2) has been widely associated with increased virulence, transmissibility, and a higher prevalence of MDR and XDR TB [18,19,20]. In China, the Beijing genotype strains have become increasingly dominant and are often linked to resistance to first- and second-line drugs [21,22,23]. Although *pncA* mutations are not genotype-specific and occur in both Beijing and non-Beijing strains [24], identifying the genotype of PZA^R^ isolates helps clarify resistance patterns and supports targeted public health efforts and molecular diagnostics.

In this study, we performed comprehensive phenotypic and genetic analyses of Mtb clinical isolates collected from TB patients to investigate the obscured mechanisms of PZA resistance in strains, which generally escape detection by conventional *pncA*-based targeted sequencing methods. Our findings aim to improve the accuracy of PZA resistance detection and contribute to the development of more reliable molecular diagnostic assays for clinical application.

## 2. Materials and Methods

### 2.1. Collection and Drug Susceptibility Testing (DST) of Mtb Clinical Isolates

A total of sixty clinical isolates were randomly collected from Guangzhou Chest Hospital, the central hospital for TB treatment in southern China. Mtb species was confirmed via Ziehl–Neelsen staining and MPB64 monoclonal antibody assay (GENESIS, Hangzhou, China) [25]. The DST of fifty-three confirmed Mtb isolates against first- and second-line anti-TB drugs was performed on Löwenstein Jensen (LJ) medium using the proportion method, in accordance with established guidelines of the WHO [1,26]. PZA susceptibility testing was carried out using the Bactec MGIT 960 system (BD-Biosciences, Sparks, MD, USA), following the manufacturer’s instructions.

Briefly, two 1 µL loops of bacterial colonies were suspended in 3 mL of phosphate-buffered saline (PBS) in a small glass tube containing glass beads. The bacterial suspension was vortexed to homogenize it and sonicated in a water bath to disperse clumps, followed by settling for 20 min. Subsequently, 2 mL of the supernatant was transferred to a fresh tube and allowed to sediment for another 15 min. After adjusting the bacterial suspension to a McFarland 0.5 standard, the suspension was further diluted 1:5 in PBS, as recommended by the manufacturer. Then, 0.5 mL of this diluted suspension was inoculated into each MGIT PZA culture tube (100 μg/mL, pH 5.9). The study protocol was approved by the Ethics Committee of Guangzhou Chest Hospital.

### 2.2. PZase Activity Assay

PZase activity was determined using the Wayne PZase test [15], with slight modifications. Briefly, 3–4 pure and freshly grown colonies were scraped off from LJ medium and transferred into 200 μL MiddleBrook 7H9 (Becton, Dickinson and Company, Franklin Lakes, NJ, USA) supplemented with albumin–dextrose–catalase (ADC) and PZA (100–300 μg/mL) in a 1.5 mL Eppendorf tube and incubated overnight at 37 °C in a shaker. Later, 15 μL of 2% Fe^2+^ was added to this 1.5 mL tube and incubated at 4 °C for 2–4 h to observe the color conversion. The positive PZase activity was detected with the appearance of a brownish color, whereas no change in color indicated negative PZase activity. Mtb H37Rv (PZA^S^) and *M. bovis* BCG (PZA^R^) served as the positive and negative controls, respectively.

### 2.3. Amplification and Sequencing of PZA Resistance-Associated Genes

Genomic DNA was extracted from freshly grown Mtb colonies using the MagMAX Total Nucleic Acid Isolation Kit (Ambion, Life Technologies, Carlsbad, NY, USA) as directed by the manufacturer. PZA resistance-associated customary genes for molecular diagnosis (*pncA*, *panD*, *rpsA*, and *Rv2783c*), including around ± 200 bp from their 5ʹ upstream and 3ʹ downstream flanking regions, were amplified in all Mtb isolates using the primers listed in Table 1 [12]. PCR amplicons were examined on agarose gels, purified using a PCR purification kit (Qiagen, Hilden, Germany), and sequenced at the BGI Genomics (Beijing Genomics Institute, Guangzhou, China). The sequences were analyzed using BioEdit v7.2.6.1 and compared to the Mtb H37Rv reference genome (NC_000962.3).

### 2.4. Whole-Genome Sequencing (WGS) and Bioinformatic Analysis

The genomic DNA extracted by the SDS method was quality-checked via agarose gel electrophoresis and quantified using Qubit fluorometry. Sequencing libraries (~350 bp in-sert size) were prepared and subjected to paired-end sequencing (PE150) on the Illumina HiSeq 2000 platform at Novogene (Beijing, China). Raw reads were filtered and base-called using CASAVA software-2.0.12, yielding FASTQ files containing sequencing and quality data. High-quality reads were aligned to the Mtb H37Rv reference genome (NC_000962.3) using the Burrows–Wheeler Aligner with the BWA-MEM algorithm. SAMtools-1.20 was used for alignment analysis and variant calling, including single-nucleotide variants (SNVs), insertions, and deletions (InDels < 50 bp). Structural variants (SVs), including large insertions, deletions, inversions, and translocations, were identified using BreakDancer. Circos was used for the visualization of coverage and variation, while CNVnator v0.3 was used to detect copy number variations (CNVs). Library construction, sequencing, and downstream bioinformatics analyses were performed using in-house pipelines and tools at Beijing Novogene Bioinformatics Technology Co., Ltd. (Beijing, China). Quality control of paired-end reads was also conducted using proprietary in-house software. Synonymous mutations were excluded to focus on potentially functional variations related to PZA resistance.

### 2.5. Validation of Novel Mutations in clpC1

To confirm novel resistance-associated mutations identified by WGS, the full-length *clpC1* gene (2547 bp), along with ~200 bp upstream and ~100 bp downstream flanking regions, was amplified and sequenced from H37Rv (NC_000962.3), PZA^S^, and PZA^R^ strains.

### 2.6. ClpC1P1P2 Proteolytic Activity Assay

ClpC1P1P2 proteins were expressed and purified following the methodology described in our recent study [27]. Proteolytic activity was assessed using 96-well white plates. Each reaction mixture of 100 µL contained 2 µM ClpP1P2, 1 µM ClpC1, and 2.5 µM fluorescein isothiocyanate (FITC)-labeled casein in the presence of POA or Ilamycin E (ILE) at a concentration of 200 μg/mL. Fluorescence was measured (excitation: 485 nm; emission: 535 nm) using a multimode plate reader at 37 °C. All reactions were performed in triplicate.

### 2.7. Detection of Beijing and Non-Beijing Genotypes

The multiplex PCR method was employed to distinguish Beijing and non-Beijing genotypes, as previously described [12]. Beijing genotype strains were identified by the absence of the genomic region spanning *Rv2816*–*Rv2819*, including part of *Rv2820*. To detect Beijing genotypes, a set of primers, namely BJ-F (5′-ACCGAGCTGATCAAACCCG-3′) and BJ-R (5′-ATGGCACGGCCGACCTGAATGAACC-3′), was used to amplify a 239 bp PCR product containing a region-specific part of *Rv2819* and part of *Rv2820*. Another pair of primers, namely NBJ-F (5′-GATCGCTTGTTCTCAGTGCAG-3′) and NBJ-R (5′-CGAAGGAGTACCACGTGGAG-3′), was used to detect non-Beijing genotypes by amplifying a 539 bp region from the *Rv2819* gene, indicative of non-Beijing strains. PCR products were analyzed via agarose gel electrophoresis.

### 2.8. Structural and Statistical Analyses

Besides the application of Bioinformatics tools for WGS data analysis, PyMOL was employed to indicate the mutational changes through in silico mutagenesis using UniPort entry P9WPC9 · ClpC1_MYCTU (https://www.uniprot.org/uniprotkb/P9WPC9/entry#sequences). Pearson’s Chi-square test was used to evaluate associations between PZA resistance and genetic/phenotypic characteristics. The diagnostic sensitivity, specificity, accuracy, and 95% confidence intervals (CIs) of genotypic testing were determined using the MEDCALC diagnostic test statistical calculator (https:/www.medcalc.org/calc/diagnostic_test.php).

## 3. Results

### 3.1. DST Profiles of Mtb Clinical Isolates

Among the fifty-three confirmed Mtb clinical isolates, twenty-four (45.2%) were identified as MDR, thirteen (24.5%) were XDR, and sixteen (30.1%) exhibited random patterns of drug resistance (Table S1). The high proportion of MDR and XDR strains is likely attributed to the source of the isolates, which were collected from a TB hospital that served as a referral center for TB patients. As this hospital receives cases from various regions, the observed prevalence of MDR and XDR TB may not be representative of the overall regional burden of drug-resistant TB. Using the gold-standard Bactec MGIT 960 system (BD-Biosciences, Sparks, MD, USA), thirty-one isolates (58.4%) were identified as resistant to PZA, while twenty-two isolates (41.5%) remained PZA-susceptible (Table S1).

### 3.2. Genotyping of PZA-Resistant Isolates

Genotyping using a multiplex PCR-based method showed that 83.01% (44/53) of the Mtb isolates belonged to the Beijing genotype, while the remaining 16.9% (9/53) were classified as non-Beijing genotypes. PZA^R^ isolates predominantly (74.1%; 23/31) belonged to the Beijing genotype, whereas 25.8% (8/31) of the PZA^R^ isolates were from the non-Beijing genotype family of Mtb strains.

### 3.3. Genetic Characterization and Association of Mutations with PZase Activity

Comprehensive genetic analysis targeting *pncA*, *rpsA*, *panD*, and *Rv2783c*, including approximately 200 bp of upstream and downstream flanking regions, was conducted to ensure complete coverage of potential resistance-associated variants [28]. We identified mutations exclusively within *pncA* or its upstream regulatory region in twenty-six PZA^R^ strains (26/31; 83.8%) (Table 2). Most interestingly, five (5/31; 16.1%) of the tested strains did not have any mutation in *pncA*, *rpsA, panD*, and *Rv2783c*, despite exhibiting phenotypic resistance to PZA.

**Figure 1:**
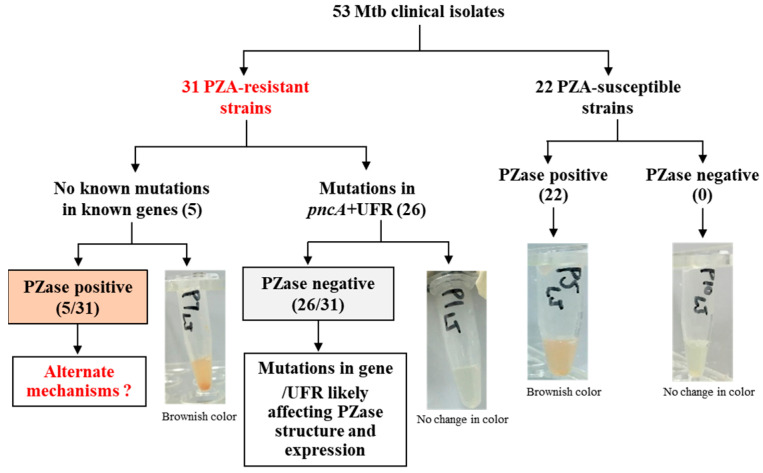
PZase activity assay of Mtb clinical isolates. The tubes in the figure present the results of the PZase activity test. In these tubes, brownish liquid indicates positive PZase activity, reflecting the hydrolysis of PZA to POA, while colorless liquid (no change in color) indicates negative PZase activity, suggesting the absence of this enzymatic function.

To assess the effect of mutations, a PZase activity assay was performed. All twenty-six PZA^R^ isolates harboring mutations in *pncA* or its upstream region demonstrated negative PZase activity, while a brownish color appeared in five PZA^R^ strains carrying wild-type *pncA*, indicating the positive PZase activity of these strains (Figure 1).

### 3.4. Identification of Novel Mutations in clpC1 of the Sequenced PZA^R^ Strains Using WGS

To resolve the observed discrepancy between phenotypic and genetic characteristics, WGS was performed on the five PZA^R^ isolates exhibiting wild-type customary genes (*pncA*, *rpsA*, *panD*, and *Rv2783c*). Comparative genomic analyses against the reference strain Mtb H37Rv (NC_000962.3) and genomic databases revealed that five PZA^R^ Mtb clinical strains contained novel nonsynonymous mutations in the *clpC1* gene, encoding an ATP-dependent ATPase involved in protein degradation.

**Figure 2:**
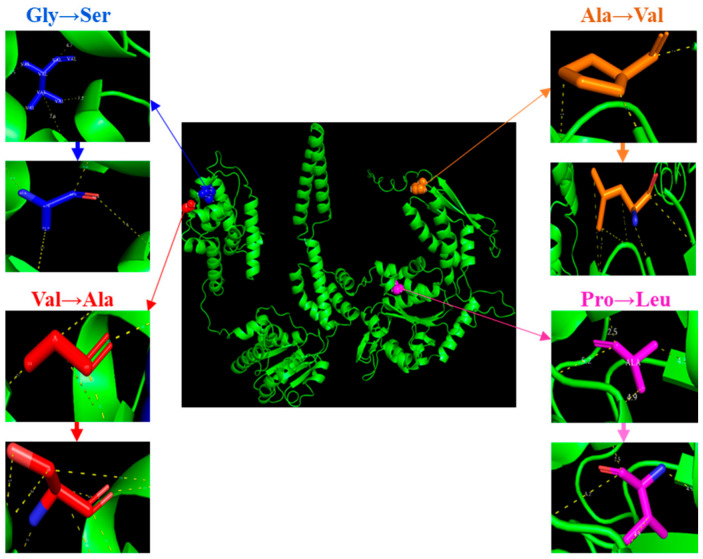
Structural changes resulting from amino acid substitutions via in silico mutagenesis of ClpC1 of Mtb.

This resulted in a clinically significant condition in which approximately 16.1% of the PZA^R^ Mtb isolates were initially misclassified as PZA-susceptible based on *pncA*-specific existing primer schemes or using conventional *pncA*-targeted molecular diagnostics alone [28], potentially leading to suboptimal treatment outcomes. Notably, the reconfirmation of these five clinical isolates through phenotypic susceptibility testing using a reduced inoculum as previously described [29,30] and targeted gene resequencing by the Sanger method verified the PZA resistance (400 μg/mL) and the existence of novel mutations in *clpC1*, respectively. In *Mtb*, ClpC1 works with the proteolytic domains ClpP1 and ClpP2 of the Clp complex to degrade misfolded proteins, a process dependent on ATP hydrolysis by ClpC1, but protein degradation is diminished or entirely halted when lacking functional ClpC1. The in silico mutagenesis by PyMOL showed structural alteration associated with these amino acid substitutions in the ClpC1 protein (Figure 2).

### 3.5. Assessment of ClpC1P1P2 Proteolytic Activity

**Figure 3:**
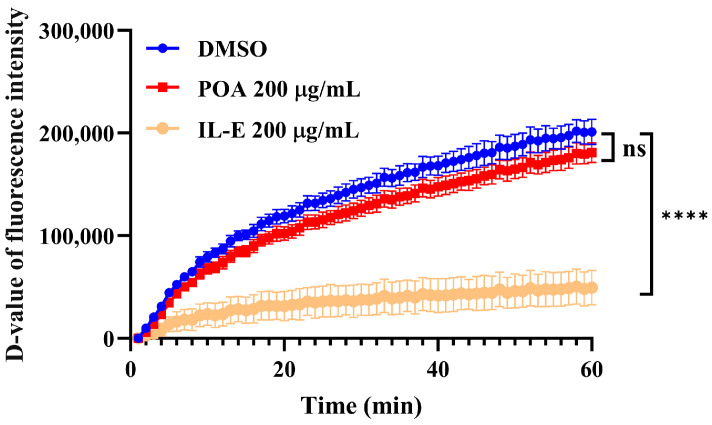
Proteolytic activity of the ClpC1P1P2 complex in response to POA treatment. **** *p* < 0.00001; ns, no significant difference, *p* > 0.05. The D-value, representing the change in fluorescence intensity, was calculated by subtracting the initial fluorescence intensity from the fluorescence intensities of subsequent detections.

It has been demonstrated that POA promotes the degradation of PanD via the ClpC1P1P2 protease complex and that mutations within ClpC1’s NBD D1 domain can inhibit this process [17]. To further investigate whether POA directly affects ClpC1-mediated proteolysis, we employed FITC-casein hydrolysis assays. While the ClpC1 inhibitor Ilamycin E (ILE) significantly suppressed ClpC1P1P2 proteolytic activity, the addition of POA had no observable effect on the degradation of FITC-casein (Figure 3), suggesting that POA does not directly inhibit ClpC1P1P2 protease activity.

### 3.6. Comparative Assessment of Molecular Versus Phenotypic Diagnostic Methods

Comparison with phenotypic DST revealed that molecular diagnosis relying solely on *pncA* sequencing exhibited a sensitivity of 48.3% with an accuracy of 69.8%. However, the detection of flanking region mutations in conjunction with *pncA* (*pncA* + FR) improved the diagnostic sensitivity to 83.8% with a concomitant increase in accuracy to 90.5%. Notably, with the identification of resistance-conferring novel mutations by WGS analysis, the sensitivity of molecular diagnosis was enhanced to 100%, and ultimately, 100% accuracy was achieved in the molecular diagnostic approach (Table 3).

## 4. Discussion

PZA, as an important first-line anti-TB drug, has unique sterilizing activity in the therapeutic regimens against persistent Mtb [3,13]. Considering its crucial role in treatment therapies, accurate detection of PZA resistance is vital for the effective inclusion of PZA in treatment regimens [12,31]. Consistent with previous studies, the rate of PZA resistance (58.4%) in our study is comparable to those reported in Hunan (60.4%) [32], Ningbo (59.1%) [33], and Myanmar (58.9%) [34]. These rates exceed those documented in South Korea (31.5%) [35], Beijing (38.3%) [36], Peru (47.7%) [37], and Ireland (55.0%) [30]. While these figures are based on clinical isolates collected from central TB hospitals or organizations and may not fully represent the general population-level prevalence, they nonetheless provide valuable insight into the regional burden of PZA resistance. Such comparative data contribute to a better understanding of resistance trends and highlight the urgent need for rapid and reliable detection methods to strengthen MDR-TB management strategies.

PZA resistance is more frequently observed in re-treated TB cases [11], underscoring the necessity for precise susceptibility testing, especially given the critical role of PZA in MDR-TB regimens. However, routine phenotypic testing remains challenging due to the requirement for an acidic environment in culture-based assays [38]. As genetic mutations are major contributors to drug resistance in Mtb, most studies on molecular diagnostics have been based on the detection of *pncA* mutations for PZA susceptibility testing [28].

In this study, 83.8% of the PZA^R^ Mtb isolates had genetic mutations in pncA or its upstream region. Mutations leading to the loss of PZase activity are the major mechanisms leading to PZA resistance. Globally, the prevalence of *pncA* mutations among PZA^R^ Mtb isolates varies significantly: 41.2% in Nepal [39], 63.6% in Fujian [40], 74.0% in Ukraine [41], 81.9–97.4% in Chongqing [42,43], 89.5% in Slovenia [44], and 95.9% in Hunan [32]. Furthermore, the dominance of the Beijing genotype (74.1%) among PZA^R^ isolates in our study mirrors findings from other studies reporting a higher frequency of MDR- and XDR-TB outbreaks caused by Beijing family strains [36,45]. These comparative data highlight the widespread involvement of *pncA* mutations in PZA resistance across different geographic regions and lineages. Such insights are important for global surveillance and the development of robust molecular diagnostics that can detect PZA resistance regardless of lineage background.

Importantly, a subset of PZA^R^ Mtb isolates in our study lacked mutations in *pncA* and other conventional resistance-associated genes (*rpsA*, *panD*, and *Rv2783c*), posing a significant diagnostic challenge for *pncA*-based molecular tests. To validate these findings, repeated phenotypic susceptibility testing using a reduced inoculum [29,30] and PZase activity assays confirmed true PZA resistance, as our results remained consistent.

**Figure 4:**
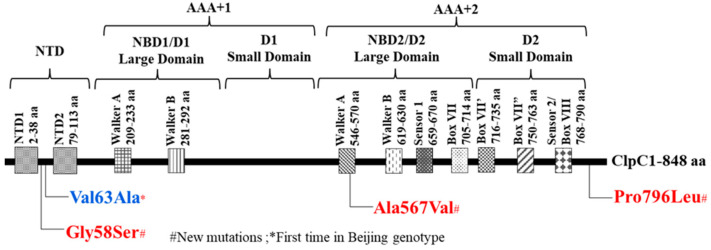
Novel mutations across ClpC1 domains of Mtb clinical isolates.

Interestingly, the WGS analysis of these five PZA^R^ Mtb isolates identified novel nonsynonymous mutations in *clpC1*, encoding an ATP-dependent chaperone ATPase crucial for protein degradation. ClpC1 belongs to the class II AAA+ family of proteins, comprising one N-terminal and two nucleotide-binding domains (D1 and D2), with the D2 domain linked to a critical C-terminal extension involved in oligomerization and substrate recognition [46,47]. The N-terminal region of ClpC1 has been recognized as a promising target site for various anti-persister drugs [48]. We identified mutations (Gly58Ser and Val63Ala) in the N-terminal domain of the ClpC1 in our PZA^R^ clinical strains (Figure 4), different from those detected at the distinct codons in the same region, causing resistance to lassomycin (Gln17Arg, Arg21Ser, Pro79Thr) [49], rufomycin (Val13, His77, and Phe80) [50], and ecumicin (Leu92Ser/Phe or Leu96Pro) [51].

Although several antibiotics share ClpC1 as a macromolecular target, the differences in their binding patterns can lead to varying effects on ClpC1 proteolytic and ATPase activities [17,50]. Moreover, the N-terminal domain of ClpC connects with adaptor proteins, either by serving as a binding site or by assisting in substrate recognition [52], suggesting that mutations in this region could interfere with substrate processing or drug binding, contributing to PZA resistance. Besides the N-terminal mutations, we also identified mutations in the D2 domain (Ala567Val) and C-terminal domain (Pro796Leu) of ClpC1 (Figure 4). The C-terminal domain has been found essential for the stability of ClpC1 hexamer and plays a critical role in protein oligomerization, which is required for its chaperone activity; however, alterations in the C-terminus lead to defects in oligomerization [47].

Owing to its crucial role in Mtb viability, ClpC1 has emerged as an attractive target for novel anti-TB agents, including rufomycin [50] and four other cyclic peptides: cyclomarin A [53], lassomycin [49], ilamycin [54], and ecumicin [51]. The anti-persister activity of these cyclic peptides through the disruption of ClpC1-mediated protein degradation is mechanistically analogous to PZA’s interference with the protein degradation system via the inhibition of trans-translation [14].

Using FITC-casein as a substrate, we confirmed that PZA’s active form, pyrazinoic acid (POA), does not directly inhibit ClpC1P1P2 proteolytic activity, unlike inhibitors such as ILE, which significantly reduced protease function [17,50,55,56]. Our data suggest that POA likely exerts its effect indirectly by promoting the degradation of PanD rather than by binding ClpC1 directly. Future studies employing detailed metabolomic and proteomic analyses are crucial to elucidate the functional consequences of ClpC1 mutations on PZA resistance mechanisms.

The increasing emergence of non-canonical resistance mechanisms that evade molecular diagnostics poses a growing challenge [57]. Mutations in clpC1 and insertions/deletions in flanking regions of pncA are often missed by conventional diagnostic assays, thereby compromising the accuracy of PZA resistance detection. According to the WHO, new molecular assays for Mtb should achieve a diagnostic sensitivity of >90% and specificity of >95% [58]. In our study, the cumulative assessment of resistance-conferring mutations demonstrated a marked improvement in diagnostic performance. The sensitivity and accuracy of PZA resistance detection increased from 48.3% and 69.8% when targeting *pncA* alone to 100% when the analysis expanded by including novel resistance markers and flanking regions. This performance surpassed the sensitivities reported in previous studies: 95.9% in Hunan [32]; 90.1% in Hangzhou [59]; 89.5% in Henan [60]; 83.1% in Ningbo [33]; 91.3% in Ireland [30]; and 97.1% in London, UK [61]. In the referenced studies, which involved comprehensive molecular analyses of PZA resistance, the most commonly examined genes besides *pncA* included *rpsA* and *panD*, which are well-established candidates associated with PZA resistance and are often included in molecular diagnostic investigations. Therefore, these genes were referred to as ‘customary genes’ in our study. Some studies have also explored additional targets such as clpC1 [17,62], Rv2783c [12], and others, albeit less consistently; however, combined evaluations of these genes assisted in increasing the diagnostic sensitivity [12]. Among them, pncA was the primary resistance determinant, with the highest frequency of mutations, particularly among the isolates belonging to the Beijing genotype. In agreement with recent reports [12,28], our findings strongly reinforce the necessity of broadening molecular diagnostic targets beyond pncA alone. Expanding the analysis by including additional resistance-associated genes and their flanking regions is essential to enhance diagnostic sensitivity and minimize the misclassification of resistant clinical isolates.

## 5. Conclusions

In conclusion, this study aims to update the growing list of PZA-associated targets by reporting the new PZA resistance-associated mutations in ClpC1 of Mtb clinical isolates. To the best of our knowledge, this is the first report from southern China that identifies novel mutations (Gly58Ser, Ala567Val, and Pro796Leu) in the ClpC1 of PZA^R^ clinical isolates (MIC ≥ 400 μg/mL) bearing wild-type *pncA*. Though Val63Ala in ClpC1 was recently stated as a low-level resistance marker in Indo-Oceanic lineage isolates [62], in southern China, it was reported for the first time in the Beijing genotype of PZA^R^ clinical isolates used in this study, which indicates that Val63Ala substitution is not lineage-specific.

Clinically, our findings advocate for the integration of long-read sequencing and parallel WGS into diagnostic workflows, particularly for isolates exhibiting phenotypic–genotypic discordance. Such approaches will not only enhance the sensitivity and accuracy of genome-based molecular diagnostics but also enable the early detection of elusive resistance mechanisms. The mechanistic investigations of our novel findings through mutagenesis in future studies will additionally pave the way for a better understanding of enigmatic resistance mechanisms and assist in the development of next-generation diagnostics to timely diagnose the elusive resistance in TB patients.

## Figures and Tables

**Table 1:** Primers for amplification of PZA resistance-associated genes.

Gene	Protein	Functional Activity	Primer Name	Oligonucleotide Sequence (5′→3′)	Product Size ~ (−200 to +200)
*pncA* (*Rv2043c*)	Pyrazinamidase/nicotinamidase (PZase)	Convert PZA into POA	pncA-F	TCGCTCACTACATCACCGGC	892 bp
			pncA-R	TCGTAGAAGCGGCCGATGGC	
* *Rv2044c + pncA* *+ Rv2042c*	Conserved hypothetical protein- PZase-Conserved protein		pncAf-F	GTGCCGCATCGAGTTCGATCCGCA	2070 bp
			pncAf-R	GATATCGGGATAGCGCCGCTGGA	
*rpsA* (Rv1630)	30S ribosomal protein S1 (RpsA)	Trans-translation	rpsA-F	ACTGAGTGCCGAGCGTGCATC	1800 bp
			rpsA-R	ACCGAACGCGTCGACCAGCG	
*panD* (*Rv3601c*)	Aspartate alpha-decarboxylase (PanD)	Pantothenate biosynthesis	panD-F	TCGACTACCTGGAGCTGCGC	755 bp
			panD-R	TCGATCGTCAGTGCCAGTTC	
*Rv2783c*	Bifunctional protein polyribonucleotide Nucleotidyltransferase (GpsI, Pnpase) and synthesize and hydrolyze (p)ppGpp	Synthesis/ degradation of ssDNA/ssRNA and (p)ppGpp	gpsI-F	ATTCAGACCTTTTCTCCTGGG	2547 bp
			gpsI-R	GTCGACTTGAACAGCAAATG	
*clpC1* (*Rv3596c*)	ATP-dependent protease ATP-binding subunit (ClpC1)	Hydrolyses proteins in the presence of ATP	clpC1-F	ACGCTTGGGTGGTTTTCTCGTT	2816 bp
			clpC1-R	ACAAACCGACGTCAGCAGAGT	

* Entire operon of *pncA* (*pncA* including flanking regions).

**Table 2:** Mutations in *pncA* and its flanking regions in PZA^R^ Mtb clinical isolates.

Locus	Nucleotide Change	Codon Change	Amino Acid Change	PZase Activity	No. of Isolates
UFR	A-11C	-	-	N	1
	A-11G	-	-	N	2
	T-122	deletion	Frameshift	N	1
	C-114, A-11G	deletion + substitution	Frameshift + substitution	N	1
*pncA* + UFR	G-115C, T476G	CTG → CGG	Leu159Arg	N	1
	C-110G, T355G	TGG → GGG	Trp119 Gly	N	1
	A403C	ACC → CCC	Thr135 Pro	N	1
	A422C	CAG → CCG	Gln141 Pro	N	2
	A-144ins, T416C	GTG → GCG	Val139Ala	N	1
	C28T	CAG → TAG	Gln10Stop	N	1
	T80G	CTG → CGG	Leu27Arg	N	1
*pncA^c^*	A128	deletion	Frameshift	N	1
	G133T	GTG → TTG	Val45Leu	N	1
	A142T	AAG → TAG	Lys48Stop	N	1
	C161A	CCG → CAG	Pro54 Gln	N	1
	C185T	CCG → CTG	Pro62Leu	N	1
	C189A	GAC → GAA	Asp63Glu	N	1
	G233A	GGC → GAC	Gly78Asp	N	2
	C282G	TTC → TTG	Phe94Leu	N	1
	A286G	AAG → GAG	Lys96Glu	N	2
	C299T	ACC → ATC	Thr100Ile	N	1
	G311T	AGC → ATC	Ser104Ile	N	1

UFR: mutations only in the upstream flaking region (UFR) of *pncA*. *pncA* + UFR: strains with mutations both in UFR and *pncA*. *pncA*^c^: mutations only in the coding region of *pncA*. N: negative.

**Table 3:** Comparative assessment of molecular versus phenotypic diagnostic susceptibility testing.

Genes	PZA^R^ Isolates n = 31		PZA^S^ Isolates n = 22		Sensitivity% (95% CI)	Specificity% (95% CI)	Accuracy% (95% CI)
	Non-Synonymous Mutations (%)	WT or Synonymous Mutations (%)	Non-Synonymous Mutations (%)	WT or Synonymous Mutations (%)			
*pncA* ^c^	15 (48.3)	16 (51.6)	0 (0.0)	22 (100)	48.3 (30.1–66.9)	100 (84.5–100)	69.8 (55.6–81.6)
UFR	5 (16.1)	26 (83.8)	0 (0.0)	22 (100)	16.1 (5.45–33.7)	100 (84.5–100)	50.9 (36.8–64.9)
*pncA* + UFR*	6 (19.3)	25 (80.6)	0 (0.0)	22 (100)	19.3 (7.45–37.4)	100 (84.5–100)	52.8 (38.6–66.7)
*pncA* + FR^#^	26 (83.8)	5 (16.1)	0 (0.0)	22 (100)	83.8 (66.2–94.5)	100 (84.5–100)	90.5 (79.3–96.8)
*clpC1*	5 (16.1)	26 (83.8)	0 (0.0)	22 (100)	16.1 (5.45–33.7)	100 (84.5–100)	50.9 (36.8–64.9)
Total	31 (100)	0 (0.0)	0 (0.0)	22 (100)	100 (88.7–100)	100 (84.5–100)	100 (93.2–100)

*pncA*^c^: mutations only in the coding region of *pncA*. UFR: mutations only in the upstream flaking region (UFR) of *pncA. pncA* + UFR*: strains having mutations both in UFR and *pncA. pncA* + FR^#^: combined strains with mutations in *pncA* and its flanking regions.

## Data Availability

The original contributions presented in this study are included in the article. Further inquiries can be directed to the corresponding authors.

## Supplementary Materials

The following supporting information can be downloaded at: https://www.mdpi.com/article/10.3390/microorganisms13061401/s1, Table S1: DST profiles of Mtb clinical isolates.
