# Supplementary material for: The Identification of Novel Mutations in ATP-Dependent Protease ClpC1 Assists in the Molecular Diagnosis of Obscured Pyrazinamide-Resistant Tuberculosis Clinical Isolates
Source: Microorganisms. 2025 Jun 16;13(6):1401. doi: 10.3390/microorganisms13061401 (PMC12196252; doi:10.3390/microorganisms13061401)
Supplement: Supplementary file 1 [file microorganisms-13-01401-s001.zip › microorganisms-3643423-supplementary.pdf]

Table S1. DST profiles of Mtb clinical isolates

Strain no.	INH	STM	RIF	EMB	AMK	LVX	MFX	PZA	Classification
TBZ1	R	S	R	S	S	R	S	S	MDR
TBZ2	R	R	R	R	R	R	R	R	XDR
TBZ3	R	S	R	S	R	R	S	S	XDR
TBZ4	R	S	R	S	S	R	R	S	MDR
TBZ5	R	S	R	R	S	R	R	S	MDR
TBZ6	R	R	R	R	S	S	S	S	MDR
TBZ7	R	S	R	R	S	R	S	R	MDR
TBZ8	R	S	R	R	S	R	R	R	MDR
TBZ9	R	R	R	R	R	R	S	S	XDR
TBZ10	R	S	R	R	S	R	R	S	MDR
TBZ11	R	S	R	R	S	R	S	R	MDR
TBZ12	R	S	R	S	S	R	R	R	MDR
TBZ13	R	S	S	R	S	R	R	S	
TBZ14	R	S	R	R	S	R	S	R	MDR
TBZ15	R	S	R	R	S	R	R	S	MDR
TBZ16	R	S	R	R	S	R	R	S	MDR
TBZ17	R	R	R	R	R	R	R	R	XDR
TBZ18	R	S	R	R	S	R	R	R	MDR
TBZ19	R	R	R	R	R	S	S	R	MDR
TBZ20	R	R	R	S	S	S	S	S	MDR
TBZ21	R	S	R	R	S	R	S	R	MDR
TBZ22	R	R	R	R	S	S	S	R	MDR
TBZ23	R	R	R	R	R	R	R	R	XDR
TBZ24	R	S	R	R	S	S	S	R	MDR
TBZ25	R	S	R	R	S	R	S	R	MDR
TBZ26	R	R	R	S	R	S	S	R	MDR
TBZ27	R	R	R	S	S	R	R	R	MDR
TBZ28	R	R	R	R	R	S	S	R	MDR
TBZ29	R	S	R	R	S	S	S	R	MDR
TBZ30	S	R	R	S	S	S	S	R	
TBZ31	R	S	S	S	S	S	S	R	
TBZ32	R	S	S	R	S	S	S	R	
TBZ33	S	S	R	S	S	R	S	R	
TBZ34	R	R	R	S	R	R	R	R	XDR

TBZ35	R	R	R	R	R	R	S	R	XDR
TBZ36	R	R	R	R	R	R	S	R	XDR
TBZ37	R	R	R	R	R	R	S	R	XDR
TBZ38	S	R	S	S	S	S	S	R	
TBZ39	R	R	R	R	R	R	S	R	XDR
TBZ40	S	S	S	R	S	R	S	R	
TBZ41	S	R	R	S	S	R	S	R	
TBZ42	R	R	R	R	R	R	R	R	XDR
TBZ43	R	S	R	S	S	R	S	S	MDR
TBZ44	S	S	S	S	S	R	R	S	
TBZ45	S	S	S	R	S	R	R	S	
TBZ46	R	S	R	R	S	R	R	S	
TBZ47	S	S	S	S	S	R	S	S	
TBZ48	R	R	R	R	R	R	R	S	XDR
TBZ49	S	R	R	S	S	R	S	S	
TBZ50	S	R	S	S	S	R	S	S	
TBZ51	R	S	S	S	R	S	S	S	
TBZ52	S	S	S	S	S	R	R	S	
TBZ53	R	R	R	R	R	R	S	S	XDR

R: Resistant, S: Susceptible, INH: Isoniazid, STM: Streptomycin, RIF: Rifampicin, EMB: Ethambutol, AMK: Amikacin, LVX: Levofloxacin, MFX: Moxifloxacin, PZA: Pyrazinamide, MDR: Multidrug-resistant, XDR: Extensively drug-resistant.
